# Autoreactive B cells in rheumatoid arthritis include mainly activated CXCR3^+^ memory B cells and plasmablasts

**DOI:** 10.1172/jci.insight.172006

**Published:** 2023-10-23

**Authors:** Sanne Reijm, Joanneke C. Kwekkeboom, Nienke J. Blomberg, Jolien Suurmond, Diane van der Woude, René E.M. Toes, Hans U. Scherer

**Affiliations:** Department of Rheumatology, Leiden University Medical Center (LUMC), Leiden, The Netherlands.

**Keywords:** Autoimmunity, Immunology, Adaptive immunity, Rheumatology

## Abstract

Many autoimmune diseases (AIDs) are characterized by the persistence of autoreactive B cell responses, which have been directly implicated in disease pathogenesis. How and why these cells are generated or how they are maintained for years is largely unknown. Rheumatoid arthritis (RA) is among the most common AIDs and is characterized by autoantibodies recognizing proteins with posttranslational modifications (PTMs). This PTM-directed autoreactive B cell compartment is ill defined. Here, we visualized the B cell response against the three main types of PTM antigens implicated in RA by spectral flow cytometry. Our results showed extensive cross-reactivity of PTM-directed B cells against all three PTM antigens (citrulline, homocitrulline, and acetyllysine). Unsupervised clustering revealed several distinct memory B cell (mBC) populations. PTM-directed cells clustered with the most recently activated class-switched mBC phenotype, with high CD80, low CD24, and low CD21 expression. Notably, patients also harbored large fractions of PTM-directed plasmablasts (PBs). Both PTM-directed mBCs and PBs showed high expression of CXCR3, a receptor for chemokines present in abundance in arthritic joints. Together, our data provide detailed insight into the biology of B cell autoreactivity and its remarkable, seemingly exhaustless persistence in a prominent human AID.

## Graphical abstract

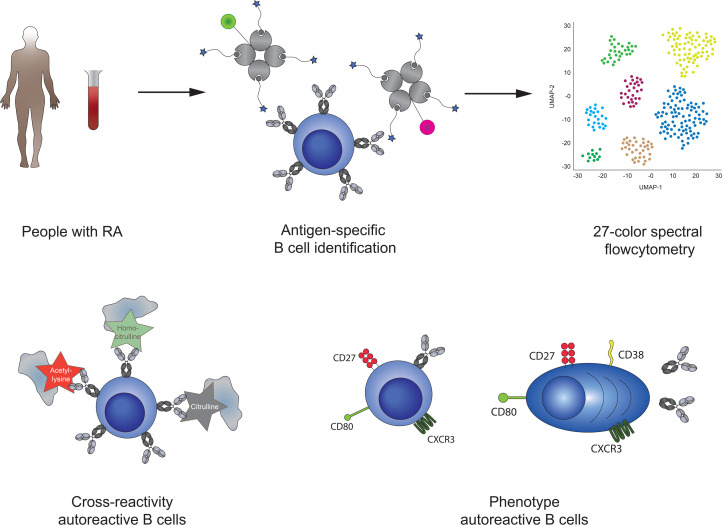

## Introduction

Many autoimmune diseases (AIDs), such as rheumatoid arthritis (RA), pemphigus vulgaris, systemic lupus erythematosus, or myasthenia gravis, are characterized by the presence of disease-specific autoantibodies. Together with the notable efficacy of B cell depletion therapy, this indicates a central role for autoreactive B cells in disease pathogenesis (1–4). Often, autoantibody responses have been well characterized. However, little information is available about the phenotype and composition of the underlying autoreactive B cell compartments. Autoreactive B cell memory frequently persists throughout a patient’s life and is difficult to eliminate therapeutically. Such lifetime memory would be desirable for vaccine responses, but information about the differences between autoreactive B cells and B cells induced by recall antigens or infections is sparse. Continuous presence, and often systemically, of antigens that immune cells can recognize is a hallmark of autoimmunity, while the presence of recall antigens is frequently transient and localized. Such different dynamics in antigen exposure can affect the outcome of immune responses. T cells exposed to immunogenic tumors, for example, lose responsiveness by expressing inhibitory molecules and/or exhaustion markers such as PD-1 and CTLA-4. Vaccine responses, on the other hand, generate memory responses that can persist for life (5). The dynamics of memory B cell (mBC) responses are, in this context, less well defined. One could, for example, expect signs of exhaustion or decay of the autoreactive mBC compartment over time, especially in instances where the autoantigen is continuously and systemically present. The observation that circulating plasma cells can differentiate from mBCs is, therefore, intriguing in an autoimmune context, as it may contain valuable clues as to how such responses can be maintained (6). In HIV infection, atypical B cells — also called double-negative 2 (DN2) or age-associated B cells — are IgD^–^CD27^–^CD21^–^ B cells that have been described to be dysfunctional or exhausted, while in SLE these B cells are linked to the production of autoantibodies (7, 8). In RA as well, atypical B cells are associated with disease, but it is unknown whether these cells are autoreactive (9). To better comprehend the biology of antigen-specific autoreactive B cell populations in humans, we visualized autoreactive B cells recognizing multiple autoantigens in RA, a prototypic AID with immunological features (such as HLA association and autoantibodies) inherent in other, less-frequent yet equally debilitating B cell–driven AIDs.

RA is characterized by systemic inflammation and a localized, destructive inflammatory process in the joints. The presence of anti–modified protein antibodies (AMPAs) is a hallmark of the disease, occurring in approximately 70% of patients. AMPA can contain reactivities to different posttranslational modifications (PTMs), including anti-citrullinated protein antibodies (ACPAs), anti–carbamylated protein antibodies (anti-CarP), and anti–acetylated protein antibodies (AAPAs). How AMPA responses are generated is currently unknown. Secreted AMPA can be highly cross-reactive to citrullinated, carbamylated, and/or acetylated antigens with varying affinities (10–12). This cross-reactivity presumably impacts B cell activation in vivo, as human B cell lines expressing B cell receptors (BCRs) recognizing citrullinated antigens can be cross-activated by carbamylated or acetylated proteins (13). The extent of cross-reactivity at the (memory) B cell level is still ill defined, however, as cross-reactivity has so far been analyzed primarily for AMPA responses in serum and for monoclonal ACPAs. It is, for example, conceivable that the secreted AMPA responses are derived from small fractions of cross-reactive PTM-directed B cells, as these could, potentially, become most activated. Insights into the cross-reactivity of PTM-directed B cells would therefore be valuable, as they could help to define the nature of PTMs to which AMPA-expressing mBCs are predominantly directed and that stimulate their activation.

Recently, we identified citrullinated protein Cit–directed (Cit-directed) mBCs in RA and revealed that these cells display an activated phenotype based on the expression of CD80, CD86, HLA-DR, and Ki-67 (14). Exploiting the possibilities of spectral flow cytometry, we now generated a combinatorial staining approach to analyze the PTM-directed plasmablast (PB) compartment alongside an extensive phenotypic evaluation of the autoreactive memory population reactive to multiple PTM antigens. Our results show the presence of a considerable population of PTM-directed PBs and point to the continuous differentiation of (activated), cross-reactive mBCs to PBs in chronic disease. This persistent activity of the PTM-directed B cell compartment may be involved in the chronicity of RA, a disease that frequently flares if antiinflammatory treatment is stopped. Hence, extensive phenotyping and improved staining methods revealed insights into PTM-directed B cell differentiation in RA, with possible implications for autoreactive B cell targeting, tolerance induction, but also efforts in the vaccine field aiming at generating long-lasting B cell memory.

## Results

### Citrullinated antigen–directed B cells are most abundant within the PTM-directed B cell population.

**Figure 1:**
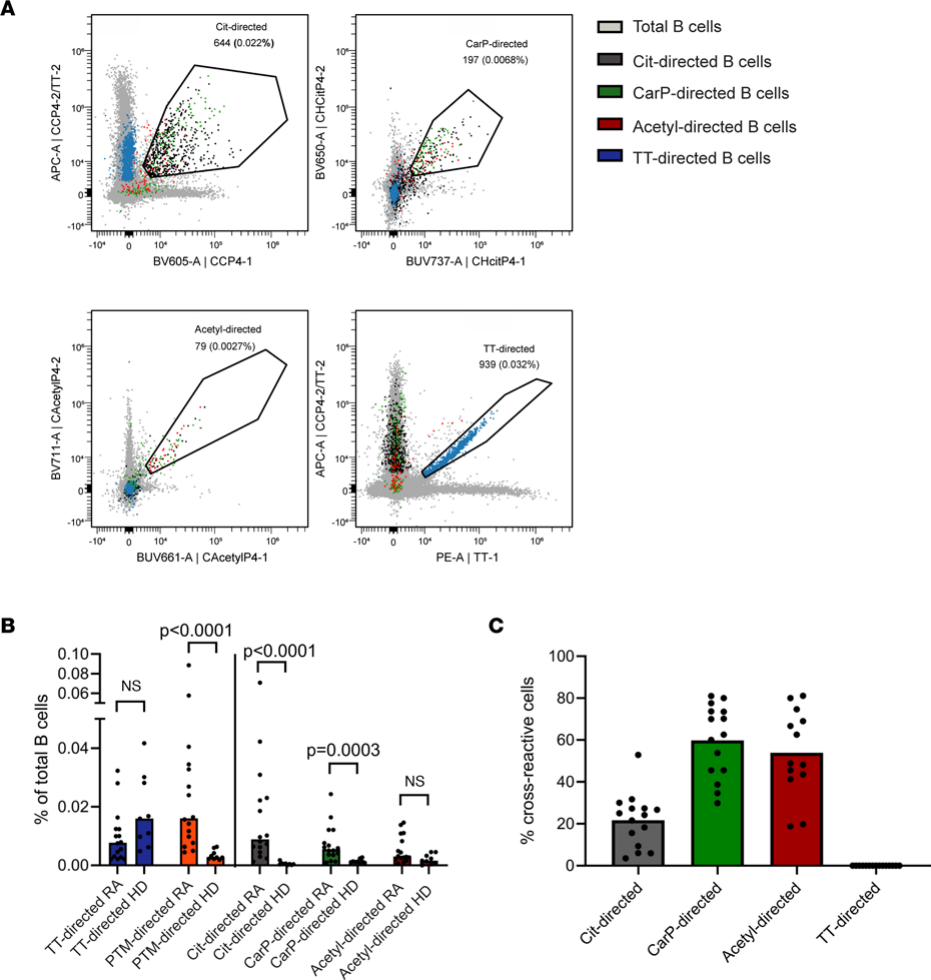
Spectral flow cytometry-based identification of PTM-directed B cells in RA patients and HDs. (**A**) Identification of Cit-, CarP-, acetyl-, and TT-directed B cells. Examples from 1 RA patient. (**B**) Quantification of PTM- and TT-directed B cells as percentage of total B cells in RA patients (*n* = 16) and HDs (*n* = 9). Each dot represents one individual; bars represent means. Statistical testing performed with Mann-Whitney *U* test. (**C**) Cross-reactivity of PTM- and TT-directed B cells in RA patients to at least one other PTM (*n* = 16). Each dot represents one individual, bars represent means.

We first set out to define the (cross-)reactivity of the PTM-directed B cell population based on the different PTM reactivities associated with RA. We visualized the different “classes” of autoantigen-specific B cells — citrullinated protein– (Cit)-, carbamylated protein– (CarP-), and acetylated protein–directed (acetyl-directed) — B cells — in RA patients by using differentially labeled streptavidin tetramers carrying individual PTM-modified peptides. Two tetramers with different fluorescent labels were generated for each modification, together with tetramers carrying control peptides with the nonmodified backbone amino acids lysine and arginine. Cells were stained simultaneously with all tetramers. This approach allowed us to obtain an antigen-specific double-staining signal per reactivity. To optimize the staining, we applied human Ramos B cell lines transduced with BCRs recognizing the different PTM antigens with predetermined cross-reactivity profiles (13). Ramos-2G9 cells stained positive for CCP4 and CHCitP4 tetramers in this setting, for example, while BCR-deficient Ramos (Ramos-MDL) cells did not show binding (Supplemental Figure 1; supplemental material available online with this article; https://doi.org/10.1172/jci.insight.172006DS1). Likewise, the acetyl-directed cell line Ramos-7E4 stained positive for CAcetylP4 tetramers. Subsequently, a panel was designed to visualize and phenotype all PTM-directed B cells. Antigens labeled to identify tetanus toxoid–directed (TT-directed) B cells and a set of markers associated with B cell activation and homing were additionally included (Supplemental Table 3). The well-defined, TT-directed B cell response was used as a comparator and control in this setting, as multiple studies by us and others have previously analyzed this response and its phenotype (14, 15). Staining B cells enriched from PBMCs from RA patients revealed that Cit-, CarP-, acetyl-, and TT-directed B cells could all be detected within the same patient and within 1 sample (Figure 1A). Of note, not all RA patients harbored B cells directed to all modified antigens. The specificity of the Cit staining was confirmed in previous studies (16), and the CarP- and acetyl-directed B cell staining was further confirmed in inhibition experiments using tetramers without fluorescent label that were added to the cells in excess before staining with the labeled tetramers (Supplemental Figure 2). The full gating strategy for 1 patient and 1 healthy donor (HD) is shown in Supplemental Figure 3. TT-directed cells were detected at similar frequencies in RA patients and HDs (Figure 1B). On the other hand, PTM-directed B cells were detected almost exclusively in RA patients, further confirming the specificity of the PTM-directed B cell staining (Figure 1B). Among the PTM-directed B cells, Cit-directed B cells were most frequent, followed by CarP-directed B cells. Acetyl-directed B cells were only detected in a minority of RA patients. Finally, a few acetyl-directed B cells could also be detected in HDs. Together, these data show the feasibility of simultaneously visualizing 3 different autoreactive B cell populations and a comparator B cell population against a recall antigen in samples from individual patients.

### PTM-directed B cells are highly cross-reactive with citrulline as dominant antigen.

Cross-reactivity within (autoreactive) B cell responses is an intriguing phenomenon, as it may point to (auto)antigens driving B cell activation beyond commonly anticipated routes. It has previously been shown that AMPAs in serum are highly cross-reactive to different PTM antigens. To understand the degree of cross-reactivity of circulating B cells, we next assessed cross-reactivity of the B cell receptors. Our results showed that a large fraction of PTM-directed B cells in the circulation reacted to multiple modified antigens, indicating that these cells were, indeed, cross-reactive (Figure 1A). More specifically, around 20% of Cit-directed B cells recognized at least 1 other PTM antigen, while the value was greater than 50% for acetyl- and CarP-directed B cells (Figure 1C). This proportion is likely an underestimation of cross-reactive cells, as the frequency of CarP-directed B cells increased when Cit- and acetyl-containing tetramers were omitted from the staining and, hence, could not compete for binding (Supplemental Figure 4). These findings indicate that a substantial proportion of modified antigen–directed cells in RA were cross-reactive to other PTMs, while others showed monoreactivity. Cit-directed B cells were most frequent and more often monoreactive than CarP- and acetyl-directed B cells, suggesting that citrulline is the dominant antigen in the AMPA response. Furthermore, these results indicate that combined staining for all 3 PTMs is required to visualize the full breadth of the PTM-directed B cell response in RA, as not all cells show cross-reactivity to other PTM antigens.

### The composition of the overall B cell population in HD and RA patients.

**Figure 2:**
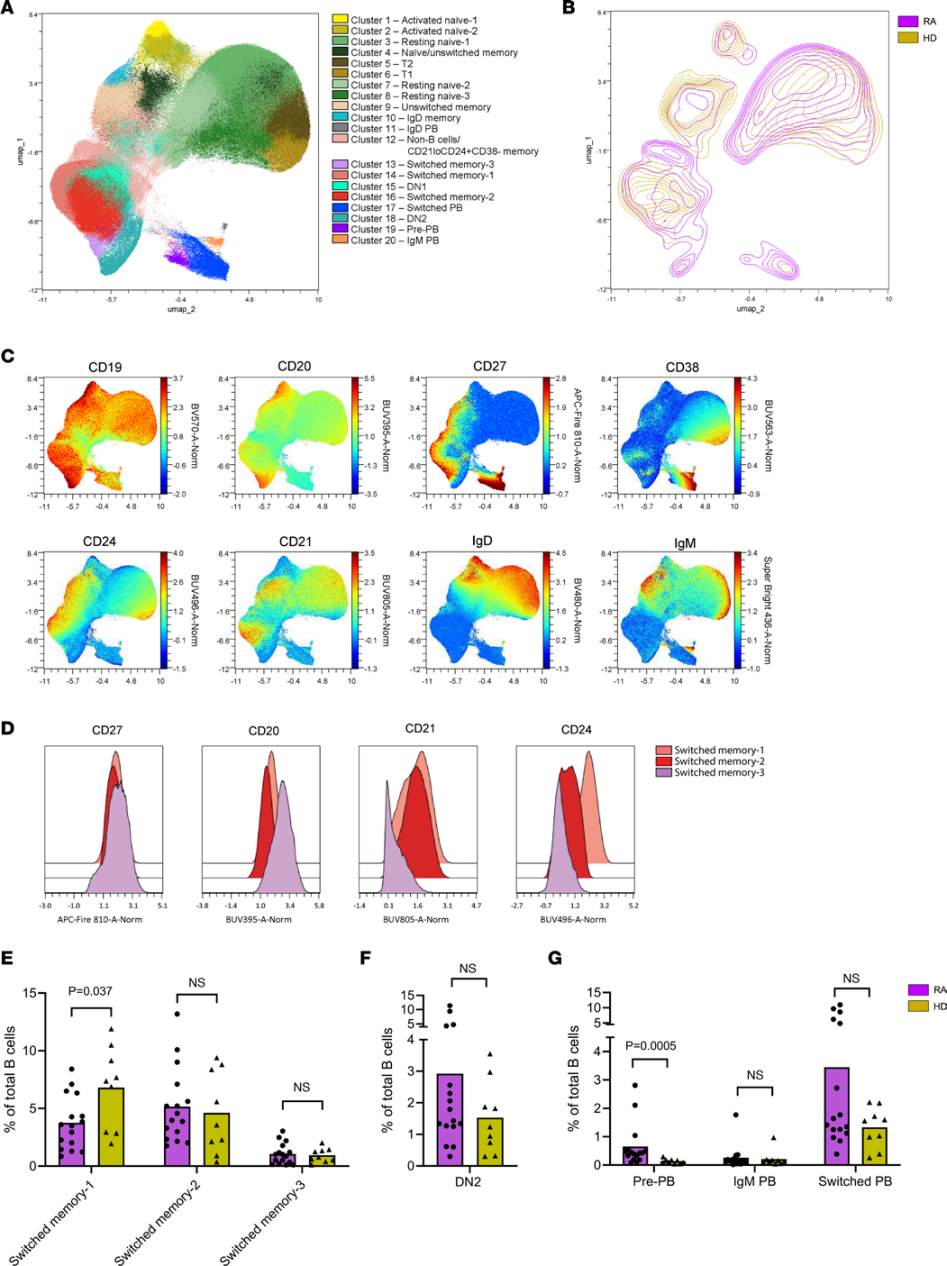
Composition and subset distribution of the B cell compartment of RA patients and HDs. (**A**) Uniform manifold approximation and projection (UMAP) visualization of B cell clusters identified by clustering on the markers represented in **C**. (**B**) Contour plot for 16 RA patients (purple) and 9 HDs (yellow) on UMAP. (**C**) Expression levels (in normalized MFI) of CD19, CD20, CD27, CD38, CD24, CD21, IgD, and IgM. Used to identify B cell clusters in **A**. (**D**) Histograms of CD27, CD20, CD21, and CD24 expression in switched memory-1, -2, and -3 subsets. (**E**–**G**) Percentages of 2 switched mBC subsets, and DN2 and PB subsets of total B cells from RA patients (purple) and HDs (yellow). Each dot represents 1 individual; bars represent means. Statistical testing was performed with Mann-Whitney *U* test.

To place the PTM-directed B cell response in the context of the total B cell population and its possible alterations in RA, we next identified B cell clusters using a self-organizing map (SOM) and normalized expression levels of the markers CD19, CD20, CD21, CD24, CD27, CD38, IgD, and IgM. This resulted in 20 different B cell clusters (Figure 2A and Supplemental Figure 5). B cell subsets were assigned names based on the expression of the markers depicted in Figure 2C and Supplemental Figure 5, according to published B cell classification (17). Interestingly, 3 different CD27^+^ switched-memory subsets were identified based on the differential expression of CD20, CD21, and CD24. “Switched memory-1” cells expressed intermediate levels of CD20 and high levels of CD21 and CD24. Switched memory-2 B cells were CD20^int^CD21^+^CD24^lo^, while switched memory-3 B cells were CD20^hi^CD21^–^CD24^–^ (Figure 2D). We found another interesting subset that clustered with PBs (cluster 19). These cells were similar to switched memory-3 cells, except that the subset lacked CD20 expression. The cells had low expression of CD62L, indicating a PB fate, but also low expression of CD38, indicating that they were not fully differentiated PBs (18). Therefore, the subset likely consisted of cells differentiating toward PBs and were called pre-PBs. To determine differences in overall B cell distribution between HD and RA patients, the B cells from these groups were visualized as depicted in Figure 2B. Differences were quantified for the switched-memory clusters, DN2 cells (CD20^+^CD27^–^IgD^–^CD21^–^), and PB clusters (Figure 2, E–G). No major differences were found between RA patients and HD in the switched memory clusters, although a small decrease for switched memory-1 was found in patients. Some RA patients showed higher percentages of DN2 cells (Figure 2F), although the difference was not significant. Furthermore, RA patients displayed a higher percentage of total pre-PBs. Finally, 5 of 16 RA patients displayed a high percentage (greater than 4% of total B cells) of switched PBs, whereas this was not found in any of the HDs.

### Recently activated IgG^+^ mBCs and a high frequency of PBs are a hallmark of the PTM-directed B cell population.

**Figure 3:**
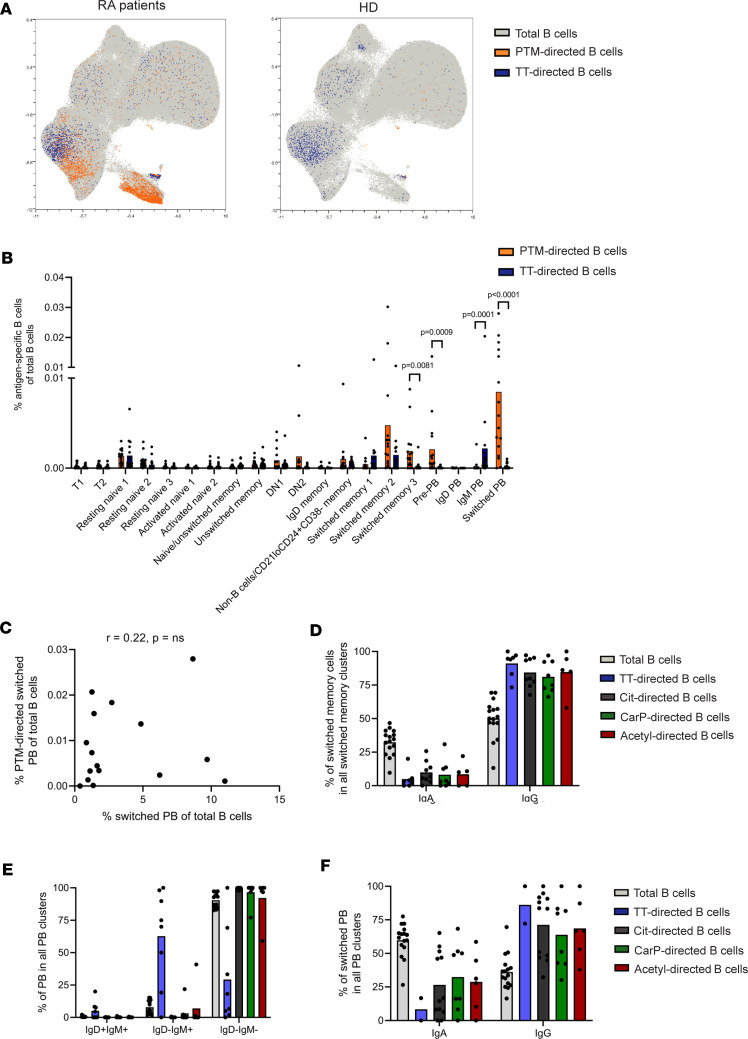
Composition and subset distribution of the antigen-specific B cell compartment. (**A**) UMAP visualization of total B cells (gray) and PTM- (orange), and TT-directed B cells (blue) from RA patients (*n* = 16) (left) and HDs (*n* = 9) (right). (**B**) Percentages of PTM- and TT-directed B cells of total B cells separated by the 20 identified B cell clusters. Each dot represents 1 individual; bars represent means. Statistical testing was performed with Wilcoxon’s signed-rank test. (**C**) Correlation between the percentage of PTM-directed switched PBs and the percentage of total switched PBs of total B cells. (**D**) Percentages of IgA- and IgG-expressing cells in the switched memory cells. (**E**) Percentages of IgM- and IgD-expressing cells in the switched PB subsets. (**F**) Percentages of IgA- and IgG-expressing cells in the switched PB subset. Each dot represents 1 individual; bars represent means. **B**–**F** include only the data from RA patients.

To further define the phenotypes of PTM-directed B cells and their non-autoreactive TT-directed comparators in this context, the same clustering strategy was applied to these antigen-specific responses. PTM-directed B cells from RA patients clustered mainly with the populations of pre-PBs, switched PBs, and class-switched mBCs (Figure 3A). Subset analysis of Cit-, CarP, and acetyl-directed B cells revealed that these responses were remarkably similar (Supplemental Figure 6). Selective expansions of autoreactive PBs directed against individual PTMs were not observed. The few PTM-directed B cells that were detectable in HDs did not cluster to a specific compartment but were scattered throughout the plot and mainly present in the resting naive B cell clusters (Figure 3A). These B cells from HDs were not included in the further analysis described below (Figure 3, B–F). TT-directed B cells, on the other hand, clustered with IgM PBs and class-switched mBCs. Interestingly, TT- and PTM-directed B cells clustered with different subsets of switched mBCs. More specifically, the highest percentages of memory PTM-directed B cells were found within the switched memory-2 and switched memory-3 clusters, while the TT-directed mBCs were almost exclusively present in switched memory-1 and switched memory-2 clusters (Figure 3B). In the PB compartment, most PTM-directed B cells were found within the switched PB cluster, but also the pre-PB cluster contained a relatively high percentage of PTM-directed cells. Of note, no correlation was found between the percentage of PTM-directed PBs and the total percentage of PBs (Figure 3C). The latter suggests that the phenotype of PTM-directed B cells was not influenced by an overrepresentation of these B cell subsets in RA patients.

Class-switch recombination is influenced by the environment in which B cells mature. Therefore, isotype use within the PTM-directed B cell compartment could provide clues about the site of B cell activation. IgA, for example, is mostly induced at mucosal sites under the influence of TGF-β (19). Within the compartment of IgM^–^IgD^–^ class-switched mBCs, the vast majority of the PTM-directed B cells and TT-directed B cells expressed IgG BCRs (Figure 3D). The PB subsets showed a pattern, as most PTM-directed PBs were class-switched (Figure 3E). IgG^+^ cells dominated the PTM-directed PB compartment, in line with their distribution within class-switched mBCs (Figure 3F). Nonetheless, there was an expansion of IgA^+^ PTM–directed B cells in about 50% of RA patients. In addition, the percentage of IgG^+^ or IgA^+^ B cells directed toward a PTM corresponded in general with plasma levels of the respective AMPAs (Supplemental Figure 7). These data suggest a close link between the PTM-directed mBCs and the PB compartment.

### PTM-directed B cells may home to sites of inflammation.

**Figure 4:**
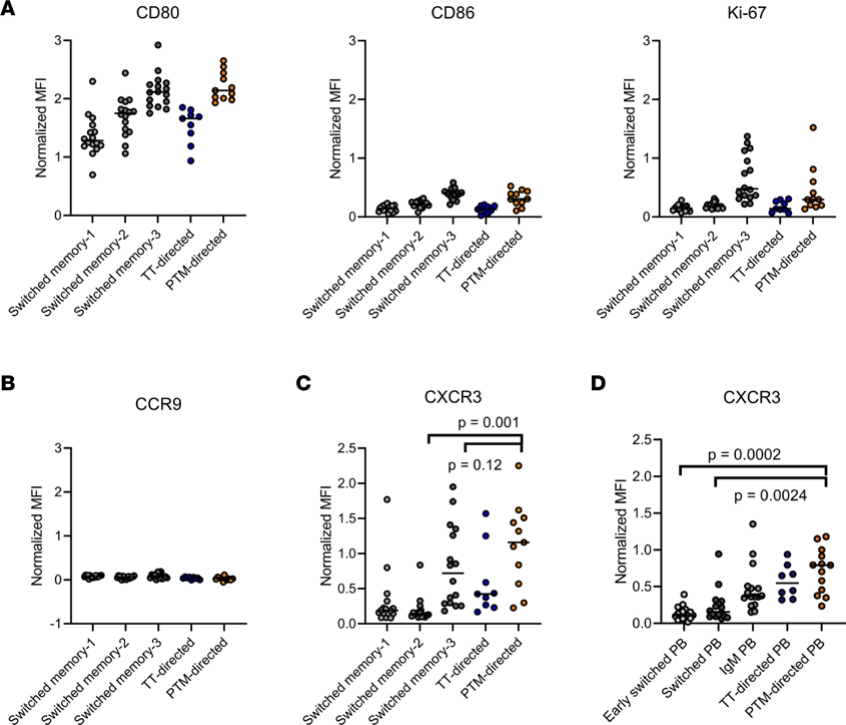
Phenotypic characteristics of PTM-directed B cells. (**A**) Normalized MFIs of CD80, CD86, and Ki-67 in 3 switched memory clusters and of TT-directed and PTM-directed B cells within these clusters. (**B**) Normalized MFI of CCR9 in the 3 switched memory clusters and TT-directed and PTM-directed B cells within these clusters. Normalized MFI of CXCR3 (**C**) in the 3 switched memory clusters and (**D**) in the 3 PB clusters, including TT-directed and PTM-directed B cells within these clusters. Differences in CXCR3 expression were statistically tested with Wilcoxson’s signed-rank test. In all plots, each dot represents 1 RA patient, and black lines represent medians. Only those PTM- and TT-directed B cells that were present in 1 of the 3 switched memory clusters (**A**–**C**) or 1 of the 3 PB clusters (**D**) were included in the analysis. Donors with fewer than 10 PTM- or TT-directed B cells within the analyzed clusters were excluded from the respective analysis.

The activation of the PTM-directed B cell compartment and the presence of a large PB fraction are intriguing. PBs are migratory cells capable of “carrying” local immune responses to distant tissues. As neither the site of generation and activation of PTM-directed B cells nor the mechanisms involved in the initiation of tissue inflammation in RA are well understood, we opted to characterize the activation and homing marker profiles of these cells. To this end, median fluorescence intensities (MFIs) of all markers were determined within the different mBC and PB clusters (Figure 4 and Supplemental Figures 8 and 9). Clustering confirmed that TT-directed and PTM-directed B cells belonged to different mBC subsets (Figure 4A and Supplemental Figure 6). Increasing expression of CD80, CD86, and Ki-67 in switched memory-1, -2, and -3 clusters, respectively, underlined that PTM-directed mBCs were among the most recently activated mBCs in peripheral blood. In contrast, TT-directed B cells remained in a resting state.

A common hypothesis about the origin of PTM-directed B cells points to mucosal tissues (20). To gather indications in support of this hypothesis, the expression of the gut-homing marker CCR9 was analyzed (19). B cells that are initiated in the gut have a higher potential to express CCR9 (21). However, CCR9 was absent on mBCs, including PTM- and TT-directed B cells (Figure 4B). IgM^+^ PBs expressed some CCR9, as did IgM^+^ TT-directed PBs, while PTM-directed B cells did not display this marker (Supplemental Figure 9). Thus, these observations suggested that PTM-directed B cells circulating in the peripheral blood did not specifically home to the gut and might have been less likely to originate from the gut.

Interestingly, the switched memory-3 B cell cluster expressed high levels of the chemokine receptor CXCR3. Expression of this receptor tended to be even higher in PTM-directed mBCs, although this difference did not reach significance (Figure 4C). In line with this finding, CXCR3 expression on PTM-directed PBs was increased significantly compared with that in all B cells in the switched (pre)PB subsets (Figure 4D). CXCR3 expression is correlated with IgG1 isotype expression (22). As PTM-directed B cells are mainly skewed to the IgG isotype, we compared all IgG memory cells and PBs with PTM-directed IgG memory cells and PBs. Interestingly, CXCR3 expression was still elevated in PTM-directed B cells (Supplemental Figure 10), indicating selective upregulation of this chemokine receptor on these autoreactive B cells. CXCR3 mediates migration of B cells toward ligands present in inflamed tissues. Hence, PTM-directed B cells in the peripheral blood were likely responsive to chemokine gradients guiding them toward the inflamed synovium in RA patients.

## Discussion

Autoreactive B cells play a major role in many AIDs. In the absence of suitable murine models that closely reflect the anti-PTM autoreactivity observed in human RA, and considering the well-known differences between human and murine (memory) B cell populations, we here focused on elaborate phenotyping of PTM-directed B cells in human samples. Our study addresses the complexity of the autoreactive PTM-directed B cell compartment by taking into account its heterogeneity as well as its remarkable cross-reactivity. Intracellular staining with isotype-specific antibodies (identifying IgG, IgA, IgM) and labeled antigens allowed us to visualize Ig isotype expression and PTM reactivity in PBs in addition to other B cell subsets. We found that approximately 50% of circulating PTM-directed B cells were indeed PBs. The size of this compartment was considerably larger than previously anticipated (14). This was likely a consequence of the challenges of incorporating PBs into B cell panels for antigen-specific studies, which requires intracellular staining, together with their vulnerability to cryopreservation (23). All analyses here were performed on fresh cells only, using protocols that allow for the visualization of both PBs and mBCs by spectral flow cytometry. Phenotypically, PTM-directed mBCs displayed a phenotype (CD19^hi^CD20^+^CD27^+^CD21^–^CD24^–^) that is often attributed to activated mBCs. Loss of CD21 and CD24 and high expression of costimulatory factors such as CD80 indicate recent B cell activation (17, 24). Furthermore, CD27^+^CD21^–^ mBCs have been linked to cells that recently left the germinal center (25). Such cells were described to express high levels of costimulatory receptors and low CCR7, which is in accordance with the PTM-directed B cell phenotype observed in this study. These mBCs might be progenitors of PBs, as similar clones have been found within this activated mBC subset and within PBs (25, 26). Interestingly, we also found PTM-directed B cells that displayed a phenotype “in between” activated mBCs and PBs. The pre-PB population supports the notion of PB-progenitor cells. Of note, the relative frequency of autoantigen-directed PBs observed in some RA patients seems to be greater than the frequency of SARS-CoV-2 antigen-specific PBs (relative to the frequency of mBCs) that are present shortly after mRNA booster vaccination (27). In this case, the frequency of virus-specific PBs equaled the number of mBCs on day 7 after administration of a booster, but dropped considerably and rapidly thereafter. On day 14 after vaccination, PBs were virtually absent. Likewise, vaccine-induced mBCs expressing a phenotype associated with recent activation (CD21^lo^CD11c^+^) dropped more abruptly than conventional mBCs. Similar findings have been described for influenza vaccination or infection (27, 28). These data are intriguing, as they point to the notion that the phenotype of the PTM-directed B cell response resembles that of B cell responses to viral antigens shortly after vaccination. The PTM-directed B cell response, however, does not transition to a resting state, but remains continuously activated and able to form PBs without signs of exhaustion or decay. In this context, it is relevant to note that the continuous activation of autoreactive responses can likely persist for years, as some RA-patients had long-standing disease. This contrasts other situations in which it has been indicated that continuous antigenic triggering induces cellular exhaustion or decay (29, 30). Resistance of PTM-directed B cell responses to such mechanisms may be an important factor in the maintenance of disease chronicity. Likewise, all patients in our study had low to moderate disease activity at the time of sampling, indicating that PTM-directed B cell responses maintain their state of activation despite the efficient therapeutic suppression of synovial inflammation. From a clinical point of view, this is perhaps not surprising, as only 10%–15% of ACPA^+^ RA patients can successfully taper immunosuppressive medication over the course of their disease, while all others will experience a flare. It is tempting to speculate that the activated phenotype of the PTM-directed B cell response contributes to flaring, as our data indicate sustained “immunological disease activity,” i.e., the absence of immunological remission despite clinical remission induced by treatment.

In chronic infections and AIDs with persistent antigen, DN2 B cells, which are CD21^–^, are often found to arise (31). In systemic lupus erythematosus (SLE), these B cells have been shown to produce autoantibodies (8). This subset has also been found to increase in RA and to associate with joint damage (32). We observed an enrichment in DN2 cells in some but not all patients. However, no enrichment of PTM-directed B cells in the DN2 population could be noted, since the CD21^–^ PTM-directed B cells were CD27^+^. This indicates that the increase in DN2 was not directly related to autoantibody production. The notion that PTM-directed B cells are CD21^–^ was also enforced by the high expression of CXCR3, a chemokine receptor that is induced by T-bet (32, 33). Expression of CXCR3 allows for migration of cells to its ligands CXCL9, CXCL10, and CXCL11. As increased concentrations of these chemokines have been observed in synovial tissue, it is likely that the synovial microenvironment can attract PTM-directed B cells efficiently (34). Indeed, increased concentrations of ACPAs and Cit-directed PBs have been observed with high frequency in synovial fluid (14, 35).

Cit-, CarP, and acetyl-directed B cells were predominantly found in RA patients, while low frequencies of these B cells were found in HDs as part of the resting naive B cell population. This finding is in line with recent studies attributing a protective function to ACPAs and to anti-PTM responses in the context of, e.g., infections. Notably, fluctuating AMPA reactivity has been described in first-degree relatives of patients with RA, while only the generation of a persisting, class-switched AMPA response associated with disease (36). The latter raises the intriguing question as to the origin of the AMPA response. Cross-reactivity and the responsiveness to external antigens may be crucial in this context. Our study provides formal evidence that not only secreted AMPAs but also PTM-directed B cells are cross-reactive to different PTMs. This is in line with data obtained from the study of monoclonal ACPAs, which were tested for the recognition of different PTMs in ELISA (12, 13, 37). Around 75% of these antibodies recognized PTMs besides citrulline in ELISA. Interestingly, this percentage is considerably higher than the 20% observed in the present study. The difference could be explained by the experimental setup, as different PTM antigens coated separately in ELISA will also allow binding of mAbs that are of lower avidity to a specific PTM. The flow cytometry setting applied in this study, however, may more closely reflect the situation in vivo, in which differences in binding avidity and antigen density will generate competition. We observed that individual tetramers indeed reduced binding to one of the other PTMs (Supplemental Figure 4) and therefore our analysis may have underestimated the degree of cross-reactivity. Depending on the application and research question, it might be more useful to stain for these PTM reactivities separately to circumvent underestimation of reactive cells to a specific modification. However, it is notable that we observed both cross-reactive and monospecific cells. This is in line with data from crystallization studies that indicate cross-reactive and “private” recognition profiles of ACPAs (38). We observed a higher percentage of cross-reactivity in the CarP and acetyl-directed compared with the Cit-directed B cell population. These data therefore suggest that PTM-directed B cells likely have the highest affinity for citrullinated epitopes. Nonetheless, B cells will most likely be exposed to all PTMs in the body. As our data showed that modified antigen-specific B cells were cross-reactive to different PTMs, these cells can become activated by a plethora of antigens if available in sufficient abundance. This may explain their persistent state of activation, but it also shows that multiple triggers, both internal or external, may drive these PTM-directed responses in individual patients.

Finally, the phenotype of PTM-directed B cells could provide a rationale to explore novel targets for the treatment of RA. Patients with highly active mBC and PB compartments, for example, could benefit from interventions that more specifically target both compartments. The expression of CD38 but also the high expression of CD19 may be useful in this respect, given the recent advances made in depleting B cells by CD19-directed CAR T cells in several rheumatic autoimmune diseases, as well as the targeted depletion of PBs by anti-CD38 therapy (3, 39).

In conclusion, we here present a deep phenotyping approach to elucidate features of PTM-directed B cells in RA. Our work revealed that these B cells — present as distinct cross-reactive populations within the larger B cell pool — had the phenotype of recently activated mBCs and PBs. They expressed high levels of CXCR3 yet lacked markers associated with mucosal gut homing. In the context of RA, and possibly also other AIDs, the long-term persistence of such activated cells may be one of the factors that determine chronicity of disease. Elucidation of the basic principles allowing the autoreactive PTM-directed B cells to continuously respond, replicate, and differentiate without showing signs of exhaustion will likely be relevant to increasing the understanding of human AIDs, but may also have implications for vaccination strategies and cancer immunotherapy.

## Methods

### Donor samples.

Peripheral blood (45 mL) was collected from 16 ACPA^+^ RA patients visiting the outpatient clinic of the Department of Rheumatology at LUMC. Peripheral blood of 9 HDs was collected via the LUMC voluntary donor service (LuVDS).

### Antigen labeling and titration.

Biotinylated CCP4 peptides (peptides of a fourth generation of cyclic citrullinated peptides (ref. 13), peptide sequences in Supplemental Table 1) were coupled to streptavidin-APC or streptavidin-BV605; biotinylated CHcitP4 was coupled to streptavidin-BV650 or streptavidin-BUV737; biotinylated CAcetylP4 was coupled to streptavidin-BV711 or streptavidin-BUV661; and the respective unmodified backbones CArgP4 and CLysP4 were coupled to streptavidin-APC-Fire750. All coupling steps were performed by incubation overnight at 4°C. The next day, free peptides were removed using a Bio-Spin P-30 column (Bio-Rad), leaving the labeled PTM tetramers in the flow-through. TT was labeled with APC or PE using an AnaTag labeling kit (Anaspec) according to the manufacturer’s protocol. Before every experiment, the labeled antigens were titrated on MDL-AID KO Ramos cells transduced with BCRs reactive to PTM antigens (13). For CCP4 and CHcitP4, the 2G9 cell line was used, and for CAcetylP4, the 7E4 cell line was used. Labeled TT was titrated on immortalized TT-specific B cells, as previously described (14).

### Flow cytometry.

PBMCs were isolated using Ficoll-plaque gradient centrifugation and stored overnight at 4°C in RPMI + 8%FCS + GlutaMAX+ penicillin/streptomycin. The next day, B cells were enriched using EasySep B cell purification kit (STEMCELL Technologies) following the manufacturer’s protocol. B cells were then stained with Fixable Viability Dye for 30 minutes on ice in the dark. After washing twice with PBS, cells were stained with CXCR3, CCR7, and CCR9 and incubated at 37°C in the dark for 30 minutes. Next, cells were washed twice with PBS + 1% BSA and stained with CD3, CD14, CD19, CD20, CD21, CD24, CD27, CD38, CD62L, CD80, CD86, IgD, and fluorochrome-labeled antigens and incubated for 30 minutes on ice in the dark. After washing twice with PBS + 1% BSA, cells were fixed and permeabilized with FoxP3/Transcription Factor Staining Buffer Set (eBioscience). Cells were subsequently stained with IgM, IgA, IgG, Ki-67, and the fluorochrome-labeled antigens and incubated for 30 minutes on ice in the dark. After washing twice with permeabilization buffer, the cells were resuspended in PBS + 1% BSA and measured with a 5-laser Cytek Aurora flow cytometer. Reference PBMCs, pooled buffy coats from 2 healthy donors, were taken along in every experiment to adjust for variation in fluorochrome intensity in different experiments, resulting in a “normalized MFI.” The fluorochromes, clones, category numbers, and dilutions of the antibodies are provided in Supplemental Table 2.

### Statistics.

Flow cytometry data were analyzed using OMIQ software from Dotmatics. Antigen-specific B cells were gated manually per experiment. Data from different experiments were normalized with CytoNorm using pooled PBMCs from 2 healthy donor buffy coats (40). Then, unsupervised FlowSOM clustering was performed using normalized CD19, CD20, CD21, CD24, CD27, CD38, IgD, and IgM to identify different B cell clusters (17, 41). Data were exported from OMIQ, and visualization and statistical testing was performed using GraphPad Prism 9.3.1. Samples with fewer than 10 cells per group were excluded from the analysis. Differences in frequencies of antigen-specific B cells or B cell subsets between HDs and RA patients were statistically tested with Mann-Whitney *U* tests. Differences in marker expression and B cell percentages in different B cell clusters were statistically tested with Wilcoxon’s signed-rank test. A *P* value less than 0.05 was considered significant.

### Study approval.

The study was approved by the IRB of LUMC. Signed IRB-approved informed consent forms were received from all patients and HDs. Patient and HDs were sex matched, but the median age of the patient group was higher than that of the HD group, which might have influenced the immune cell composition. Patient and HD characteristics are described in Supplemental Table 3.

### Data availability.

Values for all data points in graphs are reported in the Supporting data values file; human data are available in anonymized form.

## Author contributions

SR and JK performed experiments; NJB, JS, and SR designed panels; DW, REMT, and HUS supervised the project. SR analyzed the data and drafted the manuscript. All authors provided feedback and approved submission of the manuscript.

## Supplementary Material

Supplemental data

Supporting data values

## Version 1 09/19/2023

In-Press Preview

## Version 2 10/23/2023

Electronic publication
