# Supplementary material for: Autoreactive B cells in rheumatoid arthritis include mainly activated CXCR3+ memory B cells and plasmablasts
Source: JCI Insight. 2023 Oct 23;8(20):e172006. doi: 10.1172/jci.insight.172006 (PMC10619489; doi:10.1172/jci.insight.172006)
Supplement: Supplemental data [file jciinsight-8-172006-s005.pdf]

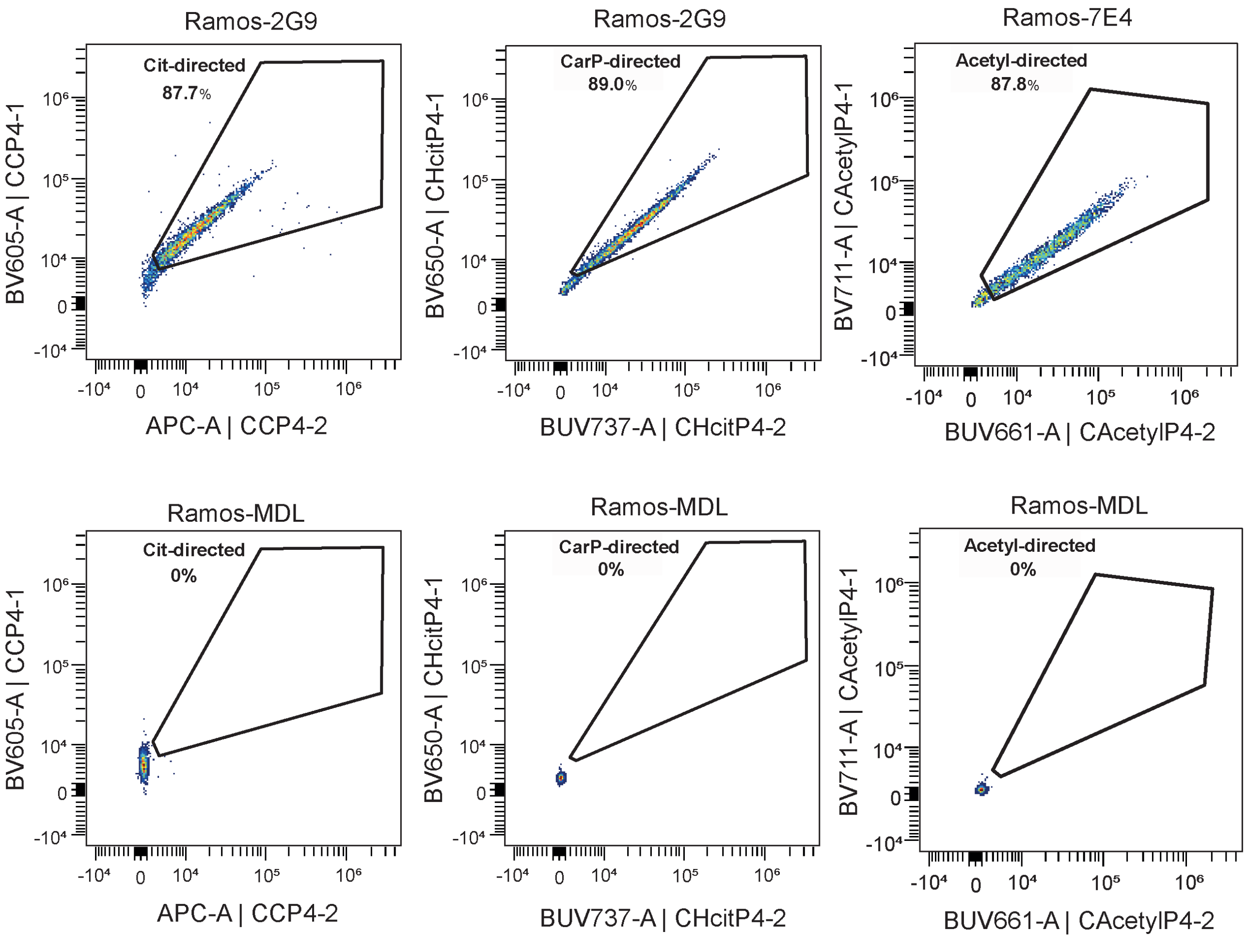

Figure S1. Tetramer staining on Ramos-2G9 or Ramos-7E4 (upper panel) or Ramos-MDL cell lines (lower panel). Cit-directed B cell staining on the left, CarP-directed B cell staining in the middle and Acetyl-directed B cell staining on the right. N = 5, one representative experiment is shown in the figure.

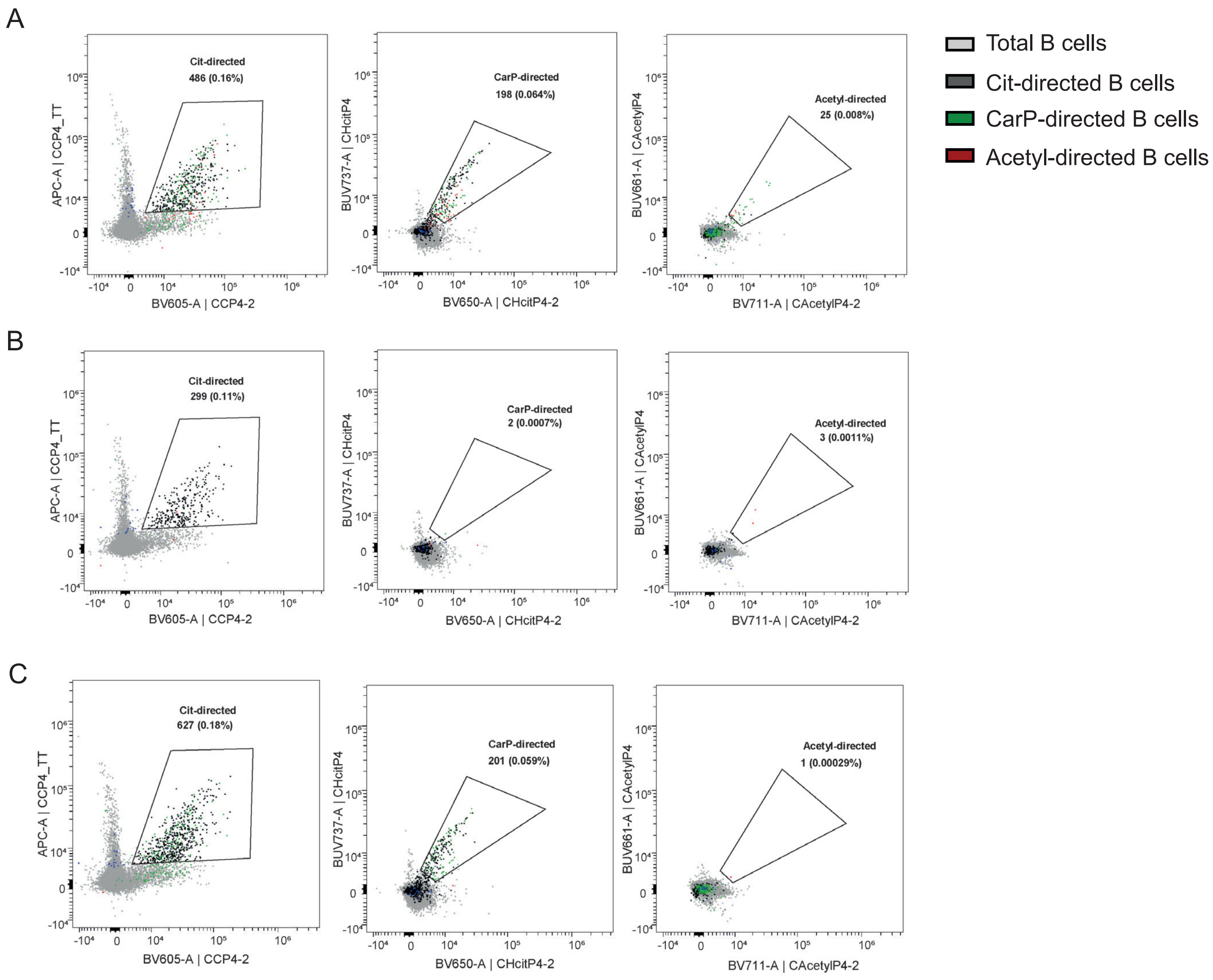

Figure S2. Blocking experiments. Cit-directed B cell staining (left), CarP-directed B cell staining (middle) and Acetyl-directed B cell staining (right) of cells from one RA patient. In A, the staining was not inhibited. In B, the signal was blocked with unlabeled CHcitP4 tetramers before staining with the labeled tetramers. In C, the signal was blocked with unlabeled CAcetylP4 tetramers before staining with the labeled tetramers.

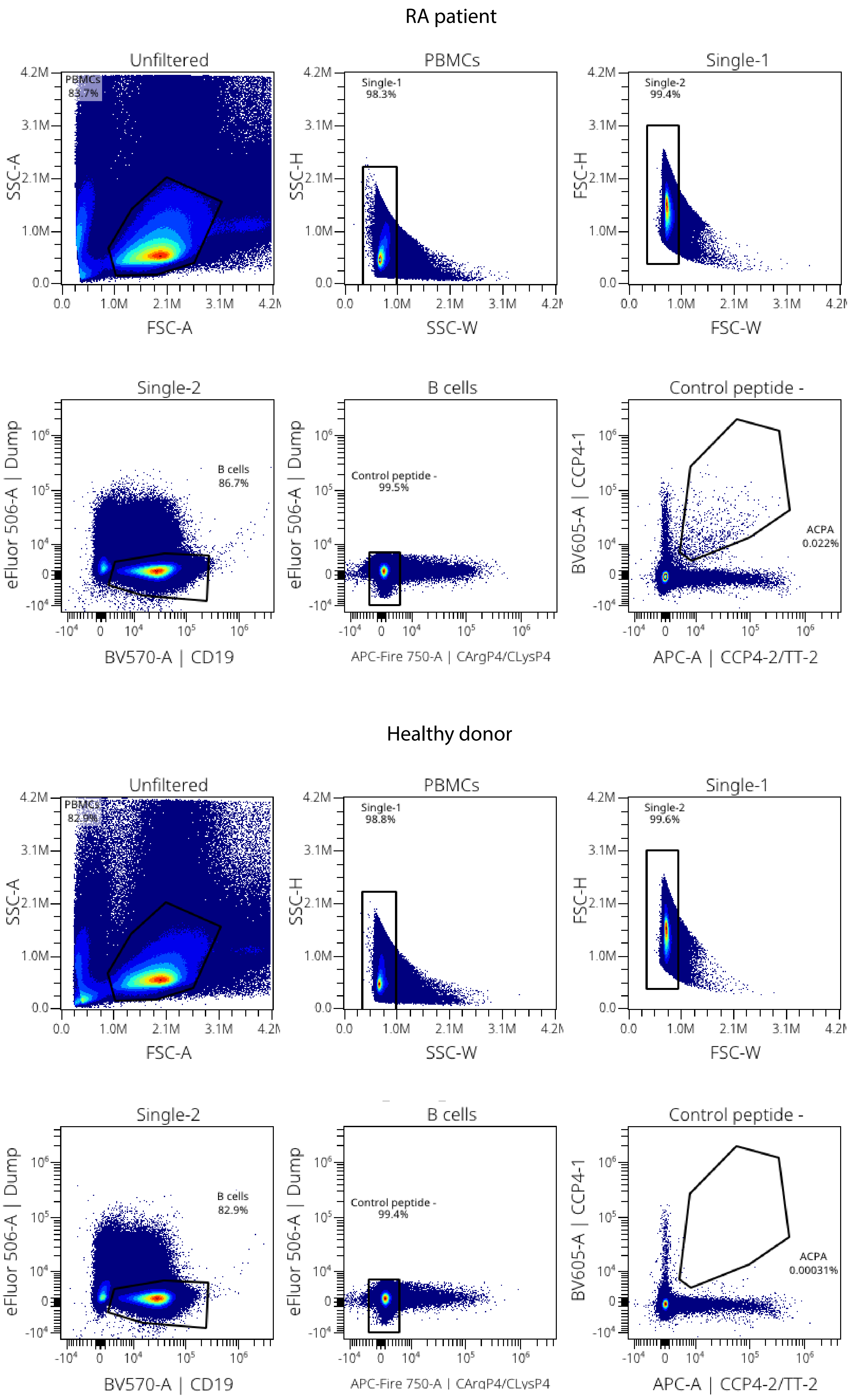

Figure S3. Gating strategy of CCP4+ B cells from one RA patient (upper panel) and one healthy donor (lower panel)

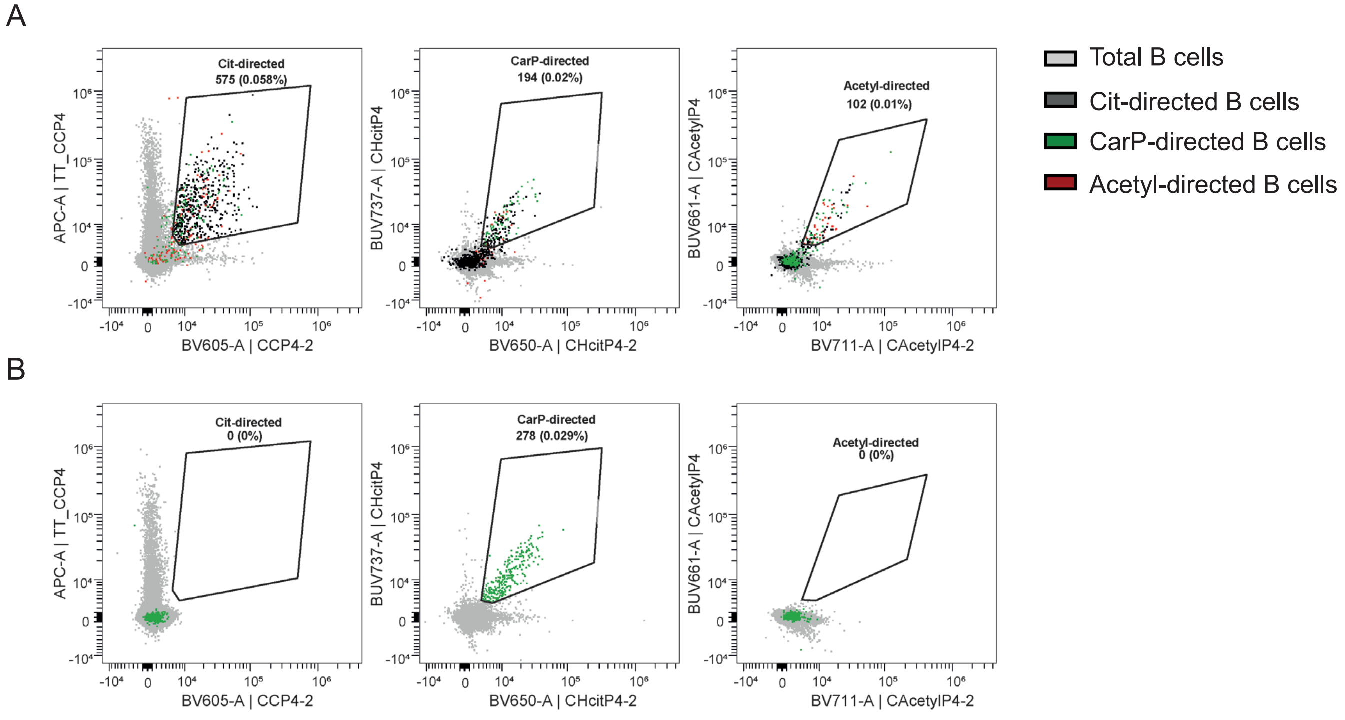

Figure S4. Cit-directed B cell staining (left), CarP-directed B cell staining (middle) and Acetyl-directed B cell staining (right) of cells from one RA patient. In A, labeled tetramers for all PTMs were added. In B, only labeled CHcitP4 tetramers were added.

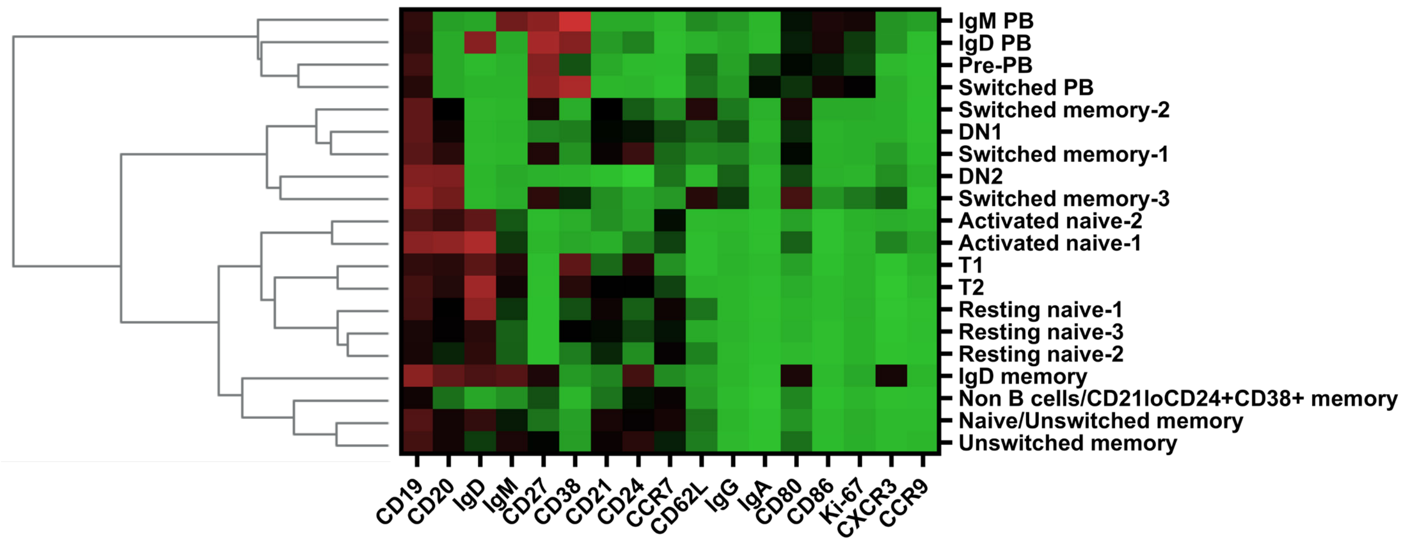

Figure S5. Heatmap with hierarchical clustering of the 20 B cell clusters obtained using the clustered heatmap function in OMIQ. From all samples (16 RA patients and 9 HD), 30,000 B cells were subsampled and the normalized MFI (green is low, red is high MFI) was used for for this analysis.

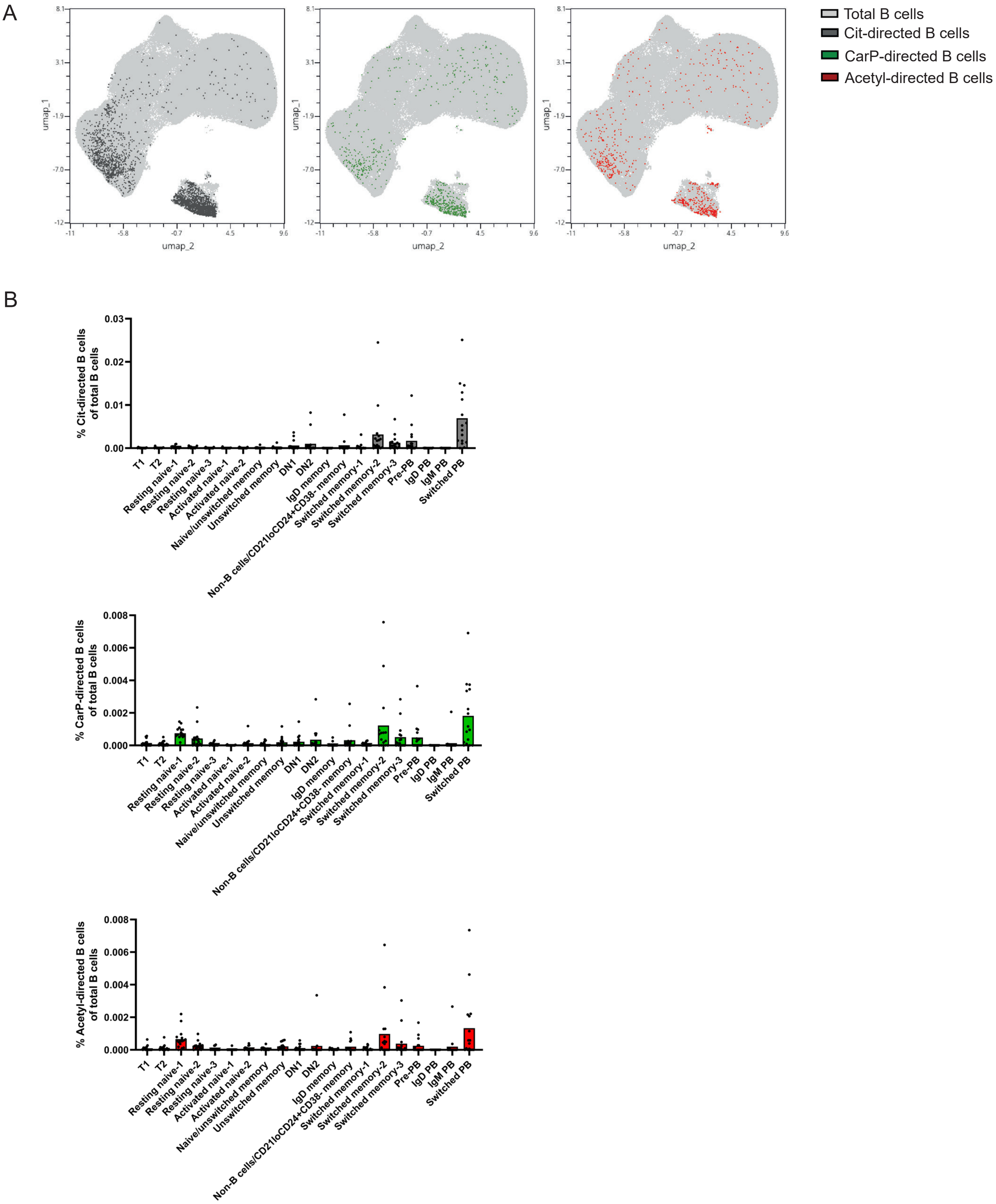

Figure S6. A) UMAP visualization of total B cells (light grey), Cit-directed B cells (dark grey), CarP-directed B cells (green) and Acetyl-directed B cells (red) of RA patients (n=16). B) Percentage of Cit-, CarP- and Acetyl-directed B cells of total B cells separated by the 20 identified B cell subsets. Each dot represents one individual, bars represent means.

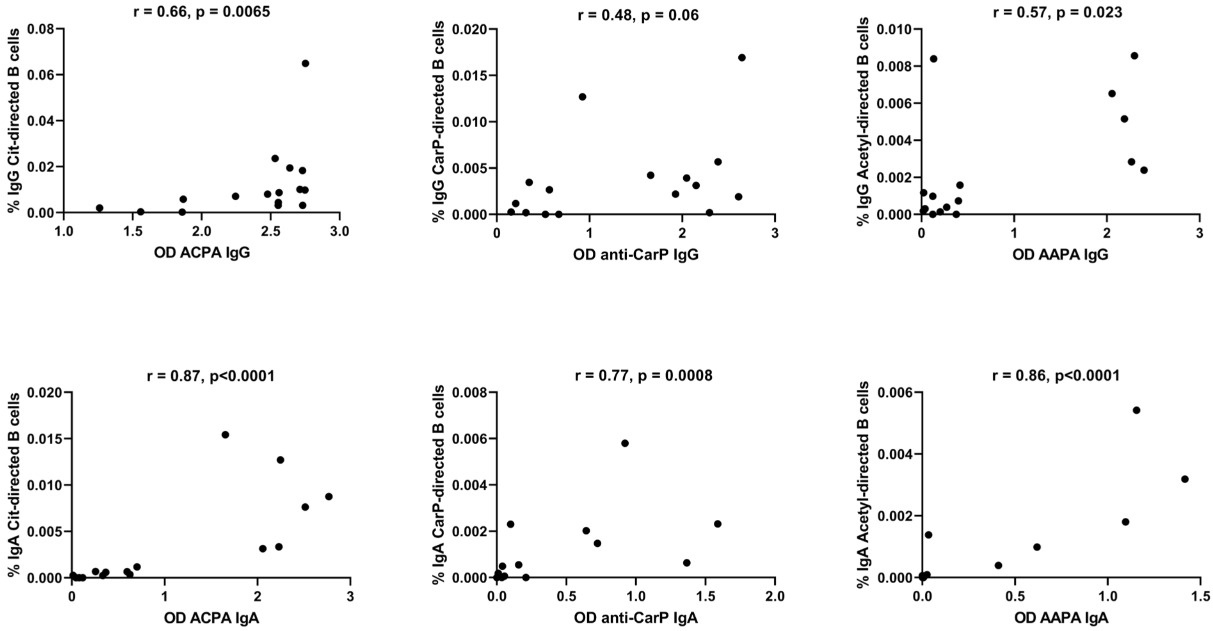

Figure S7. Correlation of the percentage of Cit-, CarP or Acetyl-directed cells of total B cells with plasma AMPA levels for IgG (upper panel) and IgA (lower panel).

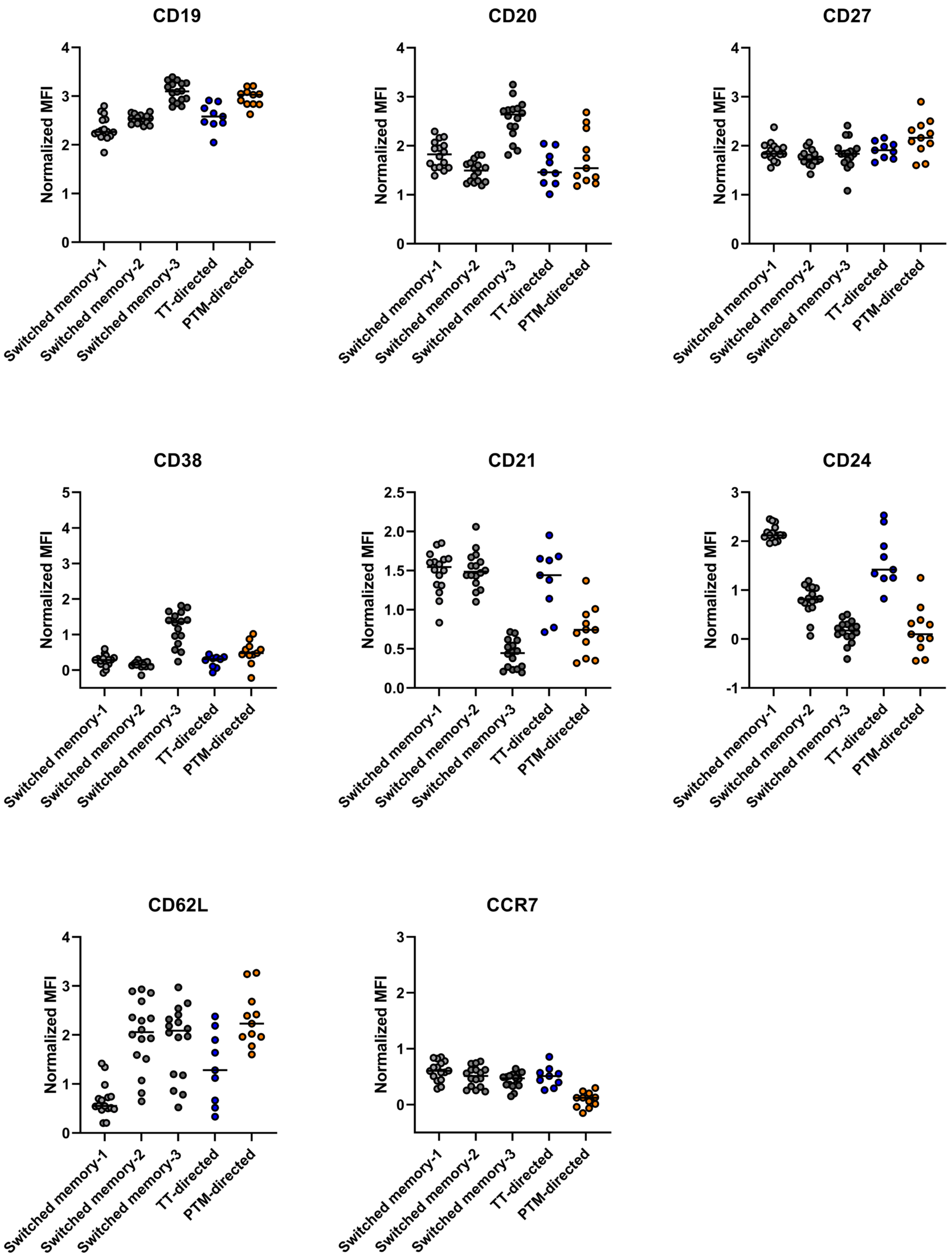

Figure S8. Normalized MFI of CD19, CD20, CD27, CD38, CD21 CD24, CCR and CD62L of 3 switched memory clusters and TT-directed B cells and PTM-directed B cells within these clusters. Each dot represent 1 RA patient. Only the PTM- and TT-directed B cells that were present in 1 of the 3 switched memory clusters were included in the analysis. Donors with less than 10 TT- or PTM-directed B cells in the analyzed clusters were excluded from the analysis.

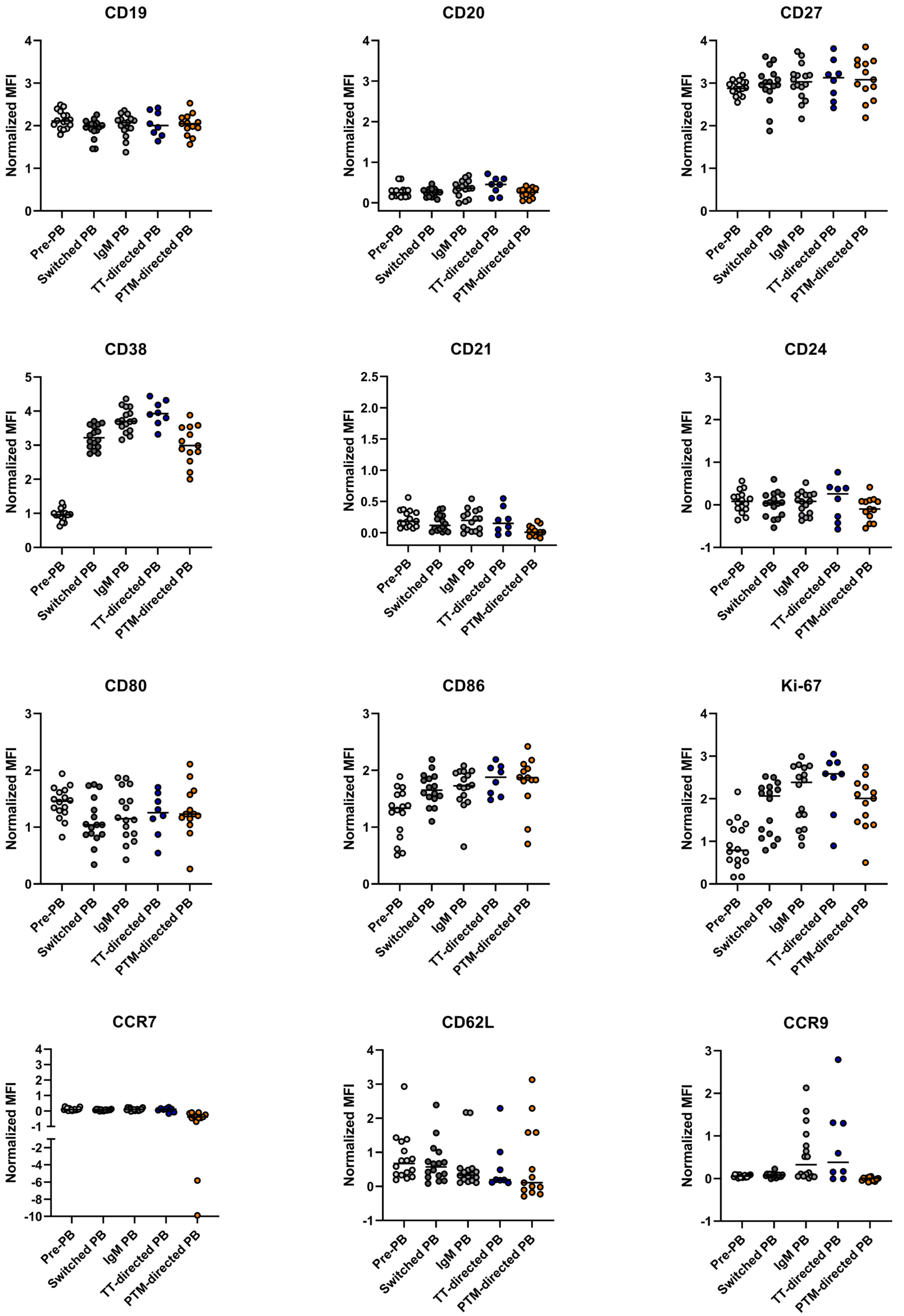

Figure S9. Normalized MFI of CD19, CD20, CD27, CD38, CD21, CD24, CD80, CD86, Ki-67, CCR7, CD62L and CCR9 of 3 PB clusters and TT-directed B cells and PTM-directed B cells within these clusters. Each dot represent 1 RA patient. Only the AMPA- and TT-directed B cells that were present in 1 of the 3 PB clusters were included in the analysis. Donors with less than 10 TT- or PTM-directed B cells in the analyzed clusters were excluded from the analysis.

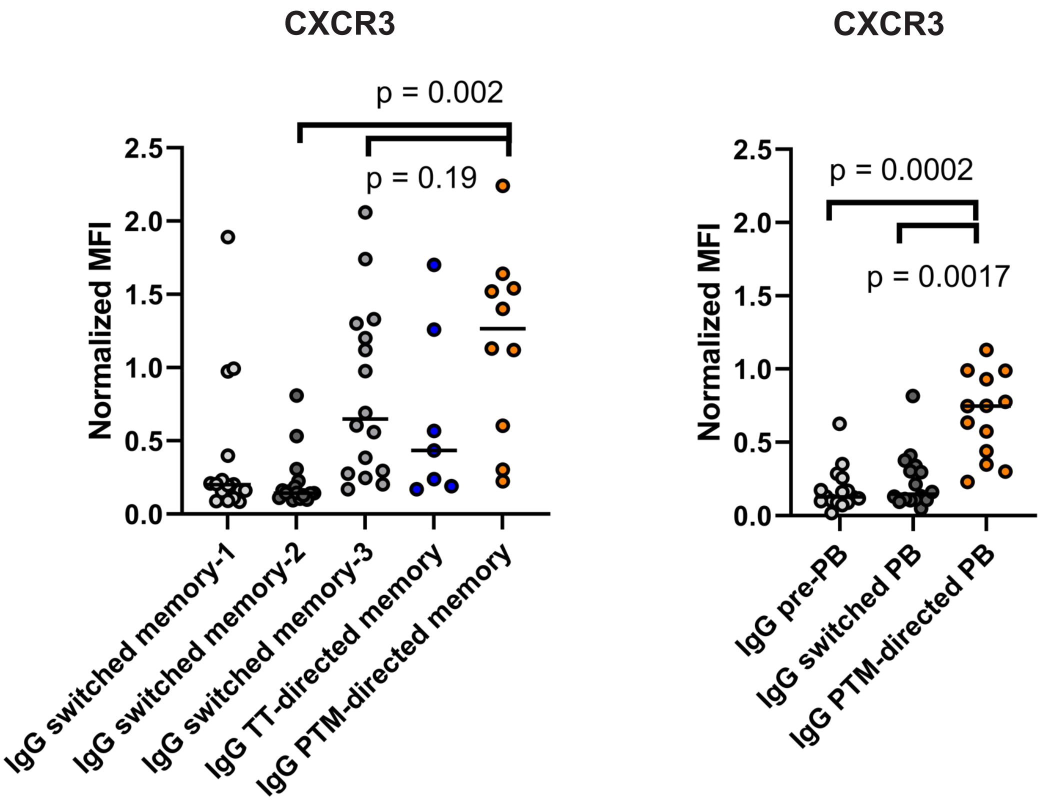

Figure S10. Normalized MFI of CXCR3 of IgG+ cells in the switched memory clusters and TT-directed B cells and PTM-directed B cells within these clusters (right). Normalized MFI of CXCR3 of IgG+ cells in the PB clusters and PTM-directed B cells within these clusters (left). Statistical testing was performed with Wilcoxon signed rank test. Each dot represent 1 RA patient. Only the PTM- and TT-directed B cells that were present in 1 of the memory or PB clusters were included in the analysis. Donors with less than 10 TT- or PTM-directed B cells in the analyzed clusters were excluded from the analysis.

Table S1 Peptide sequences

Peptide	Sequence
CCP4 (cyclic)	HQFRFXGNleSRAACZO
CArgP4 (cyclic)	HQFRFRGNleSRAACZO
CHcitP4 (cyclic)	HQFRFJGNleSRAACZO
CAcetylP4 (cyclic)	HQFRFBGNleSRAACZO
CLysP4 (cyclic)	HQFRFKGNleSRAACZO

X = citrulline
J = homocitrulline
B = acetyllysine
Z = 6-aminohexanoic acid
O = lys (biotine)-amide
Nle= norleucine

Table S2. Fluorochromes, dilutions, clones and catalog numbers of the antibodies

Antibody	Dilution	Clone	Company	Catalog
Fixable Viability Dye eFluor506	1000	n.a.	eBioscience	65-0866-14
CD3-eFuor506	25	UCHT1	eBioscience	69-0038-42
CD14-eFluor506	25	61D3	eBioscience	69-0149-42
CD19-BV570	25	HIB19	Biolegend	302236
CD20-BUV395	50	2H7	BD biosciences	563782
CD21-BUV805	400	B-ly4	BD biosciences	742008
CD24-BUV496	25	ML5	BD biosciences	741143
CD27-APCFire810	100	QA17A18	Biolegend	393214
CD38-BUV563	1000	HB7	BD biosciences	741446
CD62L-SparkNIR685	400	DREG-56	Biolegend	304861
CD80-BB515	400	L307.4	BD bioscience	565008
CD86-BV785	50	IT2.2	Biolegend	305441
CXCR3/CD183-BB700	50	1C6	BD bioscience	566533
CCR7/CD197-PE-Fire640	100	G043H7	Biolegend	353261
CCR9/CD199 -PE-cy7	800	L053E8	Biolegend	358909
IgD-BV480	400	IA6-2	BD biosciences	566138
IgA-VioBlue	400	IS11-8E10	Miltenyi	130-113-479
IgG-BV421	50	G18-145	BD biosciences	562581
IgM-SB436	50	SA-DA4	eBioscience	62-9998-42
Ki-67-PE-eFluor610	2000	20Raj1	eBioscience	61-5699-41
Streptavidin-APC	n.a.	n.a.	Invitrogen	S32362
Streptavidin-APC-Fire750	n.a.	n.a.	Biolegend	405250
Streptavidin-BUV661	n.a.	n.a.	BD bioscience	612979
Streptavidin-BUV737	n.a.	n.a.	BD bioscience	612775
Streptavidin-BV605	n.a.	n.a.	Biolegend	405229
Streptavidin-BV650	n.a.	n.a.	Biolegend	405231
Streptavidin-BV711	n.a.	n.a.	Biolegend	405241

Table S3 patient characteristics

	RA patients (16)	Healthy donors (9)
**Sex (%)**		
Male	25	22.2
Female	75	77.8
**Age (years)**		
Median	67.5	54
Range	42-87	36-66
**Most recent CCP titer (U/ml)**		
Median	>300	n.a.
Range	287-600	n.a.
**DAS28**		
Median	2.69	n.a.
Range	0.68-4.01	n.a.
**Treatment at the time of sampling**		
Methotrexate	9	n.a.
Sulfasalazine	2	n.a.
Leflunomide	1	n.a.
Anti-IL-6	1	n.a.
Anti-TNFα	2	n.a.
Prednisone	2	n.a.
Etoricoxib	1	n.a.
